# Imaging of pituitary tumors: an update with the 5th WHO Classifications—part 2. Neoplasms other than PitNET and tumor-mimicking lesions

**DOI:** 10.1007/s11604-023-01407-0

**Published:** 2023-03-13

**Authors:** Taro Tsukamoto, Yukio Miki

**Affiliations:** Department of Diagnostic and Interventional Radiology, Graduate School of Medicine, Osaka Metropolitan University, 1-4-3 Asahi-Machi, Abeno-Ku, Osaka, 545-8585 Japan

**Keywords:** Pituitary tumors, WHO classification, Diagnostic imaging, MRI

## Abstract

Many types of tumors can develop in the pituitary gland. In the recently revised 5th editions of the World Health Organization (WHO) classifications (2021 WHO Classification of Central Nervous System Tumors and the 2022 WHO Classification of Endocrine and Neuroendocrine Tumors), various changes have been made to the tumors other than pituitary neuroendocrine tumor (PitNET)/pituitary adenoma, as well as PitNET. Adamantinomatous craniopharyngioma and papillary craniopharyngioma are now considered separate tumors in the 5th edition of the WHO classification. Tumors positive for thyroid transcription factor 1, a marker of posterior pituitary cells, are now grouped together in the pituicyte tumor family in the 5th edition of the WHO classification of Endocrine and Neuroendocrine Tumors. Poorly differentiated chordoma is newly listed in the 5th edition of the WHO Classification of Endocrine and Neuroendocrine Tumors. In this paper, we present the latest WHO classification of pituitary tumors (adamantinomatous craniopharyngioma, papillary craniopharyngioma, pituitary blastoma, pituicyte tumor family, tumors of pituitary origin other than those of the pituicyte tumor family, germinoma, meningioma, chordoma, metastatic tumors, lymphoma, and pituitary incidentaloma), review diseases requiring differentiation from tumors (pituitary abscess, hypophysitis, pituitary hyperplasia, Rathke’s cleft cyst, arachnoid cyst, and aneurysm), and discuss diagnoses based on imaging findings.

## Introduction

The most common and important tumor of the pituitary gland is pituitary neuroendocrine tumor (PitNET)/pituitary adenoma; however, many other types of tumors can develop which require differentiation. Imaging, particularly magnetic resonance imaging (MRI), plays an important role in the diagnosis of pituitary diseases. The recently revised 5th editions of the World Health Organization (WHO) Classifications (2021 World Health Organization Classification of Central Nervous System Tumors and the 2022 World Health Organization Classification of Endocrine and Neuroendocrine Tumors) significantly revised the classification of pituitary adenomas, the most common pituitary tumor; entries for other tumors were also revised (Table 1) [1, 2]. This paper is the second part of a two-part series on imaging of pituitary tumors. PitNET/pituitary adenoma has been described in the part 1 [3]. This paper details the imaging findings of pituitary tumors (other than PitNETs/pituitary adenomas) and diseases that require differentiation from tumors, while considering the most recent WHO classification revisions. The imaging key points of diseases described in this two-part series are shown in Table 2.

**Table 1:** Key points in the 5th edition of the WHO classification of tumors of the pituitary region

• The most important point is the recommendation that pituitary adenoma be renamed as pituitary neuroendocrine tumor (PitNET). The International Classification of Diseases for Oncology (ICD-O) behavior code is revised from “0” to “3,” which indicates a change from benign to malignant disease. Pituitary carcinoma is also changed to metastatic PitNET
• Adamantinomatous craniopharyngioma and papillary craniopharyngioma are distinguished as separate tumor types
• Pituitary blastoma has been listed in the WHO Classification of Endocrine Tumors since the 4th edition and in the Central Nervous System WHO Classification since the 5th edition
• Pituicytoma, granular cell tumors of the sellar region, spindle cell oncocytoma, and sellar ependymoma are grouped into the pituicyte tumor family in the 5th edition of the WHO Classification of Endocrine Tumors
• Poorly differentiated chordoma *has been recognized as a subtype of chordoma with* clinicopathological features characterized by loss of SMARCB1 expression and is newly listed in the 5th edition of the WHO Classification of Endocrine and Neuroendocrine Tumors

**Table 2:** Imaging key points of pituitary tumors and tumor-mimicking lesions

PitNET/pituitary adenoma	• Signal intensity on MRI varies, because components, such as water, are not constant, and modifications, such as degeneration, hemorrhage, and infarction, also develop • Contrast-enhanced T1WI often shows mildly hypointensity compared to the normal pituitary glands • Has a later peak of contrast than the normal pituitary on dynamic MRI • Dynamic MRI is most useful in localizing microadenomas • Macroadenomas often shows a snowman shape • Macroadenomas may compress the optic chiasm and optic nerve, or involve the cavernous sinus • Macroadenomas can present as cystic masses with fluid–fluid level reflecting hemorrhage • Densely granulated somatotroph PitNET/pituitary adenoma shows hypointensity compared to the gray matter on T2WI
Adamantinomatous craniopharyngioma	• Often occurs in the suprasellar region • Typically contains both cystic and solid components • The cystic component shows hyperintensity on T1WI • The cyst wall exhibits contrast enhancement and shows annular or nodular calcification
Papillary craniopharyngioma	• 2/3 are found in the third ventricle and 1/3 in the infundibulotuberal regions • Spherical and solid with uniform contrast enhancement • Presents with a duct-like recess at the base of the mass
Pituitary blastoma	• Occurs primarily in infants • Solid tumor with a small internal cystic component • Similar presentation to macroadenoma
Pituicytoma	• Isointensity on T1WI and isointensity on T2WI to gray matter • Uniform and strong contrast enhancement • Flow voids may be prominent around the tumor
Pituitary glioma	• Grade 2 astrocytoma is common
Pituitary gangliocytoma	• Usually occurs as a mixed gangliocytoma-PitNET/pituitary adenoma • Cannot be distinguished from PitNETs/pituitary adenomas
Sellar atypical teratoid/rhabdoid tumor (AT/RT)	• Occurs in adults and mostly in women • Similar presentation to macroadenoma
Germinoma	• Found in the hypothalamus, pituitary stalk, and posterior pituitary gland • CT shows high density to gray matter • Contrast-enhanced T1WI shows a uniform contrast enhancement • DWI shows hyperintensity
Meningioma	• Purely intrasellar meningiomas are rare • Presents with a normal (compressed) pituitary gland • CT may show calcifications within the tumor, bony hyperostosis, or an enlarged sphenoid sinus (pneumosinus dilatans) in adjacent regions • Contrast-enhanced T1WI shows a uniform contrast enhancement • Dural tail is frequently observed
Chordoma	• CT shows extensive lytic bone destruction • On T2WI, conventional chordoma typically shows marked hyperintensity • Contrast-enhanced T1WI shows moderate-to-marked contrast enhancement, and may appear a “honeycomb” enhancement pattern • ADC of conventional chordomas is about 1.5 × 10^–3^ mm^2^/s Poorly differentiated chordoma often shows hypointensity on T2WI compared to conventional chordomas
Metastatic tumors	• May present as a dumbbell-shaped tumor • Sellar bone erosion may be seen but not sellar enlargement
Lymphoma	• Contrast-enhanced T1WI shows a uniform contrast enhancement • DWI shows hyperintensity • The cavernous sinus is involved in approximately 40% of cases
Pituitary abscess	• Cystic lesion within the pituitary gland • Marked hyperintensity on DWI • Contrast-enhanced T1WI shows rim-like contrast enhancement • Often shows thickening of the pituitary stalk, predominantly in the inferior region
Hypophysitis	• Symmetrical enlargement of the pituitary gland and stalk • Contrast-enhanced T1WI shows a uniform contrast enhancement • Loss of hyperintensity in the posterior pituitary gland on T1WI • Thickening of the of the surrounding dura mater (dural tail) may be observed • Hypointense regions on T2WI of the peripituitary region (parasellar T2-dark sign) may be observed
Pituitary hyperplasia	• Often shows homogeneous intensity and contrast enhancement on MRI
Rathke’s cleft cyst	• Usually occurs between the anterior and posterior lobes of the pituitary gland • Often shows hyperintensity on T1WI • Characteristic intracystic nodules reflecting waxy component (so-called waxy nodule) may be observed • The cyst wall usually has non-contrast enhancement
Arachnoid cyst	• T1WI and T2WI show a thin-walled cyst with a homogeneous signal equal to that of the cerebrospinal fluid • Arachnoid cyst within the sella turcica tends to push the pituitary gland posteriorly
Aneurysm	• CT and MR angiography aid in diagnosis • Hypointense on T2WI due to flow void • Thrombosed areas show hyper-or heterogeneously intense signals on T1WI • The intensity can be altered by calcifications, lamellated blood degradation products, and flow-related signals

*MRI* magnetic Resonance imaging; *T1WI* T1-weighted image; *T2WI* T2-weighted image; *CT* computed tomography; *DWI* diffusion-weighted imaging; *ADC* apparent diffusion coefficient

## Craniopharyngiomas

WHO classifications prior to the 5th edition categorized adamantinomatous craniopharyngioma and papillary craniopharyngioma as subtypes of the same tumor. However, the 5th edition of the WHO classification classifies these as separate types of tumors.

### Rationale for classifying adamantinomatous craniopharyngioma and papillary craniopharyngioma as separate tumors

Both adamantinomatous craniopharyngioma and papillary craniopharyngioma exhibit squamous differentiation but differ in age of predilection and imaging/histopathologic findings. Molecular genetic research recently revealed that adamantinomatous craniopharyngioma has a CTNNB1 mutation but no BRAF V600E mutation, and papillary craniopharyngioma has a BRAF V600E mutation but no CTNNB1 mutation [4]. CTNNB1 encodes β-catenin, and CTNNB1 mutations are associated with tumorigenesis via the WNT/β catenin pathway. The BRAF V600E mutation is associated with tumorigenesis via the MAPK pathway. Therefore, adamantinomatous and papillary craniopharyngiomas have different mechanisms of tumorigenesis and are thus considered separate tumors in the fifth edition of the WHO classification. These tumors are described as follows. Adamantinomatous craniopharyngioma is a mixed solid and cystic squamous epithelial tumor with stellate reticulum and wet keratin, usually localized to the hypothalamic–pituitary axis and characterized by activating CTNNB1 mutations [1]. Papillary craniopharyngioma is a solid or partially cystic, non-keratinizing squamous epithelial tumor that develops in the infundibulotuberal region of the third ventricle floor, most often in adults, and is characterized by BRAF p.V600E mutation [1].

### Adamantinomatous craniopharyngioma

#### Epidemiology

Adamantinomatous craniopharyngiomas account for approximately 3% of all intracranial tumors. A bimodal age distribution occurs with incidence peaks in children (5–15 years) and adults (45–60 years) [1]. In children, adamantinomatous craniopharyngiomas account for up to 10% of intracranial tumors, are more common than PitNET/pituitary adenomas, and are the most common saddle tumors [5]. No sex predilection is observed [1].

#### Imaging findings (Fig. 1)

**Fig. 1:**
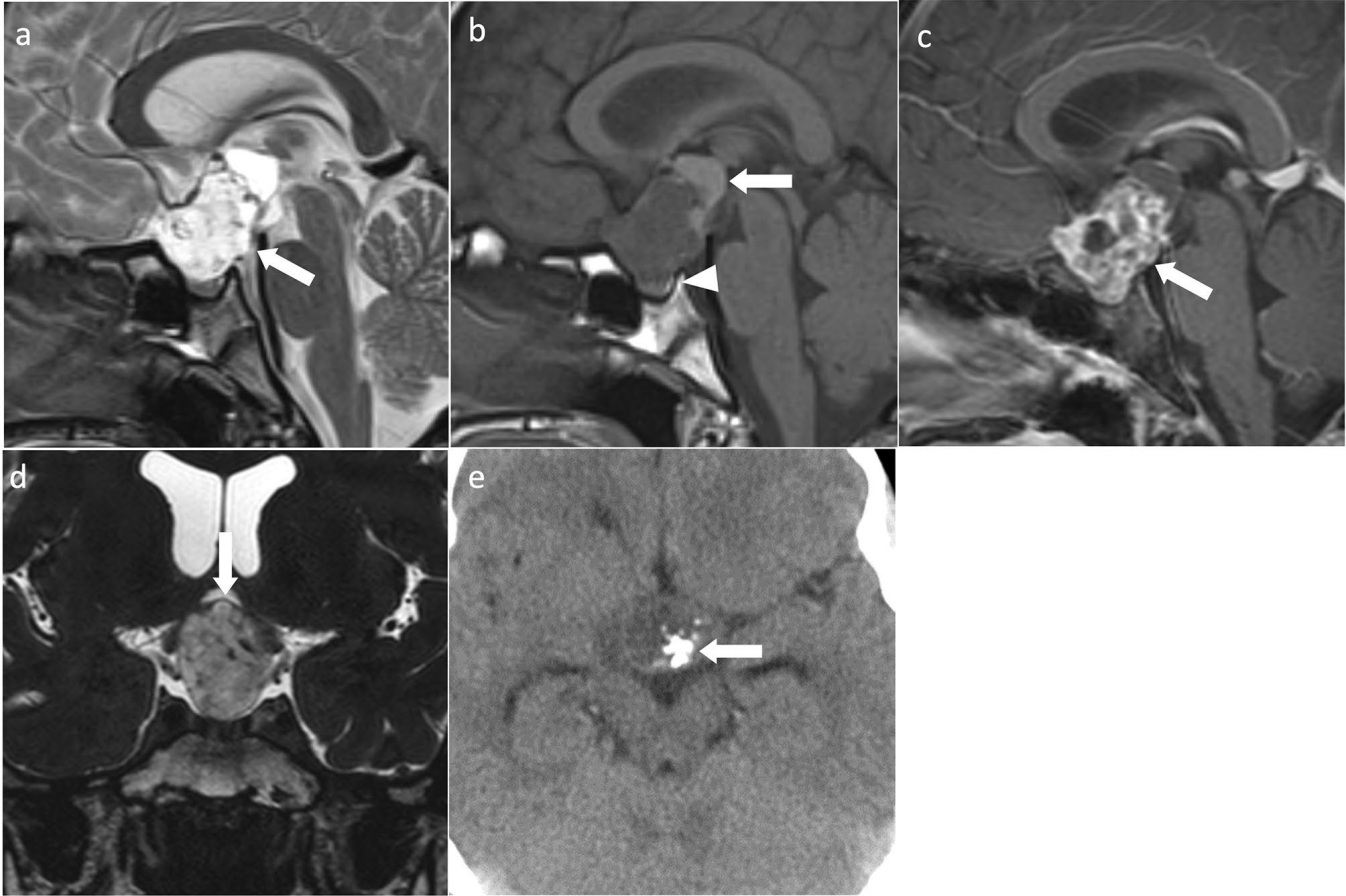
Adamantinomatous craniopharyngioma. A 13-year-old girl with headache and vision loss. **a** Sagittal T2WI shows a multifocal cystic mass above the sella turcica (arrow). The pituitary gland is observed within the sellar turcica. **b** Sagittal T1WI shows some cysts with mild hyperintensity (arrow) compared to white matter. Normal hyperintensity in the posterior pituitary gland is observed (arrowhead). **c** Sagittal contrast-enhanced T1WI shows heterogeneous contrast enhancement. The pituitary stalk is inside the mass and cannot be identified (arrow). **d** Coronal heavy T2WI shows the optic chiasm compressed by the mass (arrow). **e** Non-contrast computed tomography shows calcification inside the mass (arrow)

Adamantinomatous craniopharyngiomas often occur in the suprasellar region (> 90%) [6] and typically contain both cystic and solid components on MRI [7]. The cystic component shows hyperintensity on T1-weighted images (T1WI), reflecting secondary to high protein content, cholesterol, mild calcification, or hemorrhage [7]. The cyst wall exhibits contrast enhancement and shows annular or nodular calcification [8]. Computed tomography (CT) is useful for calcification detection. Edematous changes in the brain parenchyma along the optic tract adjacent to the mass have been observed and are considered characteristic [9]; however, these have also been reported in PitNETs/pituitary adenomas, germ cell tumors, and malignant lymphomas [10].

The differential diagnoses are PitNETs/pituitary adenomas, particularly cystic degenerated PitNETs/pituitary adenomas. Calcification is suggestive of adamantinomatous craniopharyngioma. Moreover, adamantinomatous craniopharyngioma often has a lobulated shape, third ventricle compression by superior tumor extension, mixed solid and cystic characteristics, and reticular enhancement of the solid portion. Conversely, PitNETs/pituitary adenomas have a snowman shape, solid characteristics, and homogeneous enhancement of the solid portions [11]. Quantitative textural analysis of MR images has been reported to aid differential diagnosis of pituitary PitNETs/pituitary adenomas from adamantinomatous craniopharyngiomas [12].

### Papillary craniopharyngioma

#### Epidemiology

Papillary craniopharyngioma is principally an adult disease (peak incidence: 30–59 years). A sex predilection has not been reported [1].

#### Imaging findings (Fig. 2)

**Fig. 2:**
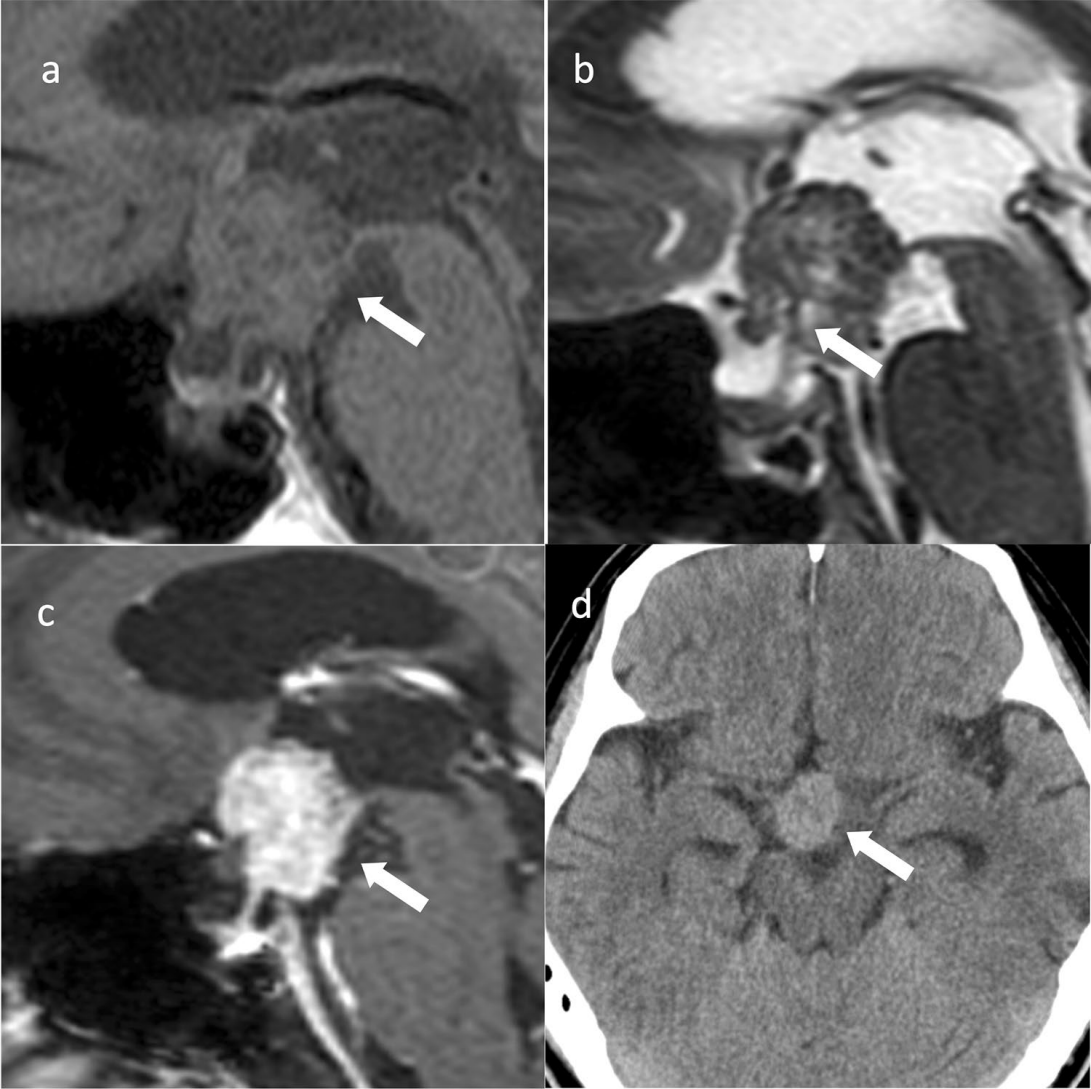
Papillary craniopharyngioma. An approximately 50-year-old male with eye floaters. **a** Sagittal T1WI shows a mass with isointensity compared to white matter from the suprasellar region into the third ventricle (arrow). **b** Sagittal T2WI shows some small cyst-like hyperintensity areas in a iso- to hypointense mass compared to white matter (arrow). A duct-like recess is observed at the base of the mass contiguous with the pituitary stalk (arrow). **c** Sagittal contrast-enhanced T1WI shows almost uniform and strong contrast enhancement (arrow). **d** Non-contrast computed tomography shows mild high density (arrow). The patient underwent surgery via nasal endoscopy, and the mass was confirmed as a papillary craniopharyngioma arising from the base of the third ventricle

Approximately two-thirds are found in the third ventricle and one-third in the infundibulotuberal regions [13]. These tumors are rarely confined within the sella turcica or suprasellar cistern. Intrasellar involvement is not common.

Most papillary craniopharyngiomas are spherical and solid with uniform contrast enhancement. Some are mixed solid/cystic or predominantly cystic. Cystic lesions often show hypointensity on T1WI [14] and have a solid, cauliflower-like nodule [1]. Papillary craniopharyngioma of the third ventricle may present with a duct-like recess at the base of the mass, differentiating it from other third ventricle tumors (sensitivity, 33%; specificity, 100%) [15]. Calcification is rare [16]. Differential diagnoses include chordoid glioma of the third ventricle, pituicytoma, and meningioma.

## Pituitary blastoma

Pituitary blastoma is extremely rare, with fewer than 20 cases [17]. Pituitary blastoma has been described since the publication of the 4th edition of the WHO Classification of Endocrine Tumors and is listed in the 5th editions of the WHO Classification of Endocrine Tumors and the WHO Central Nervous System Classification.

Pituitary blastoma is an embryonal neoplasm of the sellar region and is composed of primitive blastemal cells, neuroendocrine cells, and Rathke’s pouch epithelium. It is associated with germline or somatic DICER1 mutations and occurs primarily in infants (median age, 8 months) [18]. Symptoms are observed in Cushing’s syndrome due to adrenocorticotropic hormone (ACTH) production. Hormone production may be absent. Ocular paralysis may occur with tumor growth. DICER1 mutation-associated tumors include pleuropulmonary blastoma, cystic nephroma, ovarian Sertoli–Leydig cell tumor, ciliary body medulloepithelioma, nasal chondromesenchymal hamartoma, sarcomas of the cervix, kidneys, cerebrum, pituitary blastoma, and pineoblastoma, among others [19].

### Imaging findings

Although there have been few cases and no comprehensive imaging reports, pituitary blastoma has a similar presentation to macroadenoma and is a solid tumor with a small internal cystic component within the sella turcica and over the suprasellar region [19, 20].

## Pituicyte tumor family

Pituicytomas, granular cell tumors of the sellar region, spindle cell oncocytoma, and sellar ependymoma are positive for thyroid transcription factor 1, a marker of posterior pituitary cells. Therefore, since the 4th edition of the WHO Classification of Endocrine Tumors, they have been treated as the same group of tumors. Pituicytes, which are posterior pituitary cells, are classified into five types based on electron microscopic morphological features. Light and dark include pituicytoma, oncocytic includes spindle cell oncocytoma, granular includes granular cell tumors, and ependymal includes sellar ependymoma [21]. The 5th edition of the WHO Classification of Endocrine Tumors includes and groups pituicytoma, granular cell tumor of the sellar region/granular cell pituicytoma, spindle cell oncocytoma/oncocytic pituicytoma, and ependymal pituicytoma under the pituicyte tumor family [2]. The 5th edition of the Central Nervous System WHO classification does not use the term ‘pituicyte tumor family’ and does not list sellar ependymoma or ependymal pituicytoma [1].

### Epidemiology

Many of these tumors occur in adults in the fifth-to-sixth decades of life [22]. A slight male predilection has been reported for pituicytomas, and a slight female predilection has been reported for oncocytic and granular cell tumors [1].

### Imaging findings

**Fig. 3:**
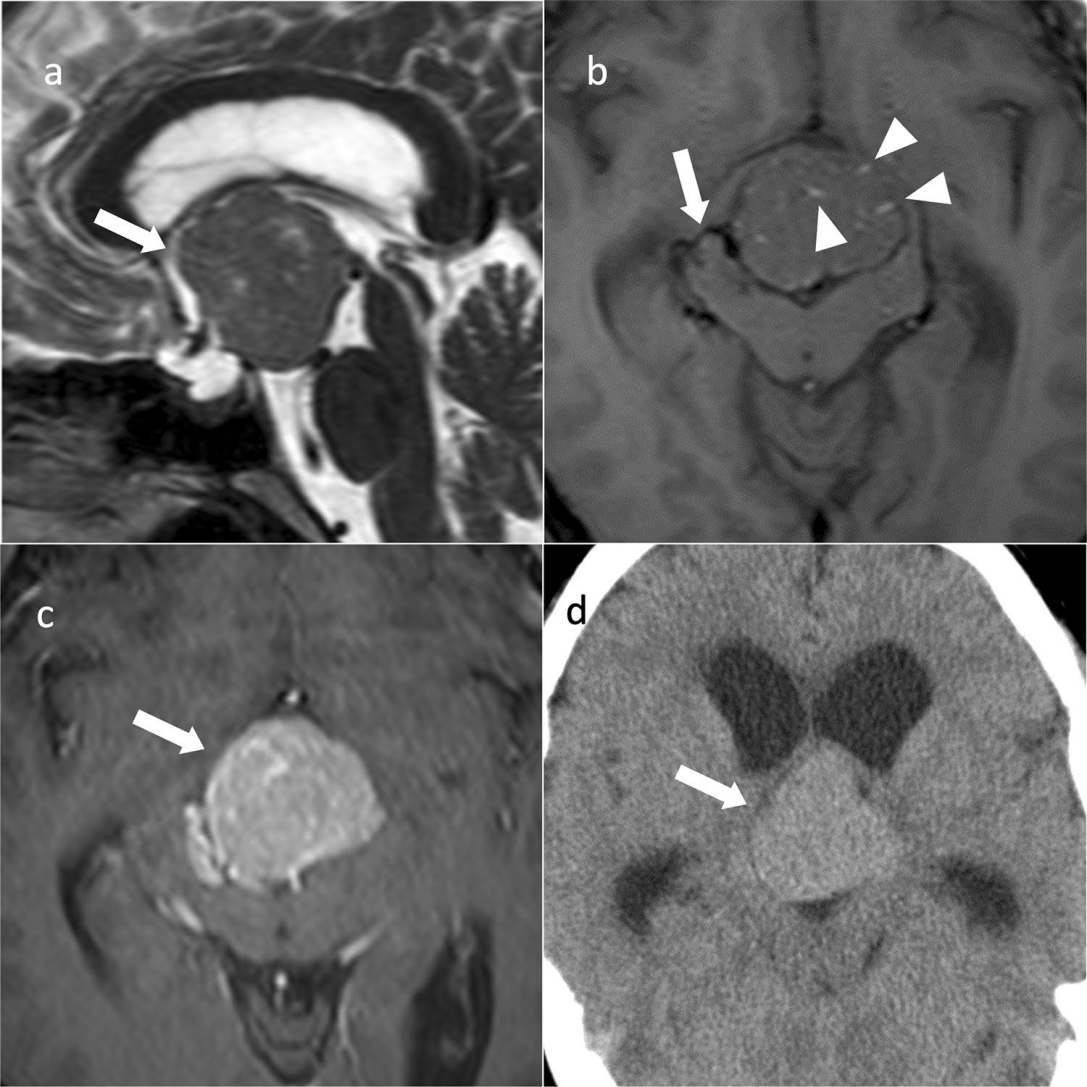
Pituicytoma. An approximately 40-year-old male with mild cognitive decline. **a** Sagittal T2WI shows a lobulated mass with isointensity compared to gray matter in the suprasellar region (arrow). **b** Axial T1WI shows a flow void around the mass (arrow). Small hyperintense areas within the mass show slow-flowing vessels (arrowheads). **c** Contrast-enhanced T1WI shows strong and uniform contrast enhancement (arrow). **d** Non-contrast CT shows mild high density (arrow). Ventricular enlargement is observed

Pituicytoma exhibits slightly high density on CT. Internal necrosis, cystic degeneration, and calcifications are rare [23, 24]. Approximately 20% are localized within the sella turcica, 40% within the intrasellar-to-suprasellar region, and 40% within the suprasellar region, with isointensity on T1WI and hyperintensity on T2WI compared to white matter, often with uniform and strong contrast enhancement. Flow voids may be prominent around the tumor (Fig. 3) [23, 24]. Dynamic contrast-enhanced MRI may show earlier time to peak of the tumor than that of the anterior pituitary gland, reflecting the blood flow in the posterior pituitary gland [23].

Imaging findings of granular cell tumors of the sellar region and spindle cell oncocytoma overlap with those of pituicytomas. Spindle cell oncocytoma has a greater invasive tendency than those of other types of oncocytoma [21].

## Tumors of pituitary origin outside the pituicyte tumor family

### Pituitary glioma

Pituitary gliomas account for 1.4% of all gliomas in the central nervous system [25] and can occur in children and adults. The average age of onset is 28 years, which is lower than that of intracranial gliomas (43 years). Grade 2 astrocytoma (regardless of IDH mutation) is common, followed by pilocytic astrocytoma [25]. High-grade gliomas are uncommon [25].

### Pituitary gangliocytoma

Pituitary gangliocytoma occurs more frequently as a mixed gangliocytoma-PitNET/pituitary adenoma than it does as an isolated gangliocytoma [26]. Occasionally, tumors have a glial component and are defined as gangliogliomas [2]. This tumor occurs almost exclusively in women [27]. Approximately 75% of patients exhibit pituitary or hypothalamic hormone hypersecretion, and this tumor cannot be distinguished preoperatively from PitNETs/pituitary adenomas [26].

### Sellar atypical teratoid/rhabdoid tumor (AT/RT)

AT/RT is a malignant tumor of the central nervous system characterized by the deletion of the SMARCB1 (INI1) gene on chromosome 22q11.2. AT/RT usually occurs in children under 3 years of age, whereas sellar AT/RT occurs in adults (median age of 44 years [range: 20–70 years]) [28] and predominantly in women (94.7%) [28]. Imaging findings overlap with those of macroadenomas. This tumor presents as a well-developed mass extending from within the sella turcica to the suprasellar region, often with heterogeneous contrast enhancement. Approximately 40% of cases involve the cavernous sinus and 20% have cystic changes [28]. Only fewer than 50 cases of sellar AT/RT have been reported, and thus, details of imaging characteristics, including CT attenuation and apparent diffusion coefficient (ADC), have not been reported [28].

## Germinoma

Germ cell tumors are more common in East Asia than in Western countries [29]. In the United States, it occurs in 3.9% of brain tumor patients under the age of 20, while in Japan, it occurs in 16.9% [29]. Germ cell tumors can be divided into germinomas and non-germinomatous germ cell tumors. Germinomas comprise 2/3 of cases, while non-germinomatous germ cell tumors comprise 1/3 of cases; 90% of cases occur in patients younger than 20 years [30]. Intracranial germ cell tumors often occur in the pineal gland and suprasellar region [30]. Other onset locations include the basal ganglia, ventricles, thalamus, medulla oblongata, and cerebral hemispheres [30]. Pineal germinomas show male predominance (male-to-female ratio, 3:1), whereas suprasellar germinomas show female predominance [30]. Both the pineal gland and suprasellar region may be involved, more commonly in males [30].

### Imaging findings

**Fig. 4:**
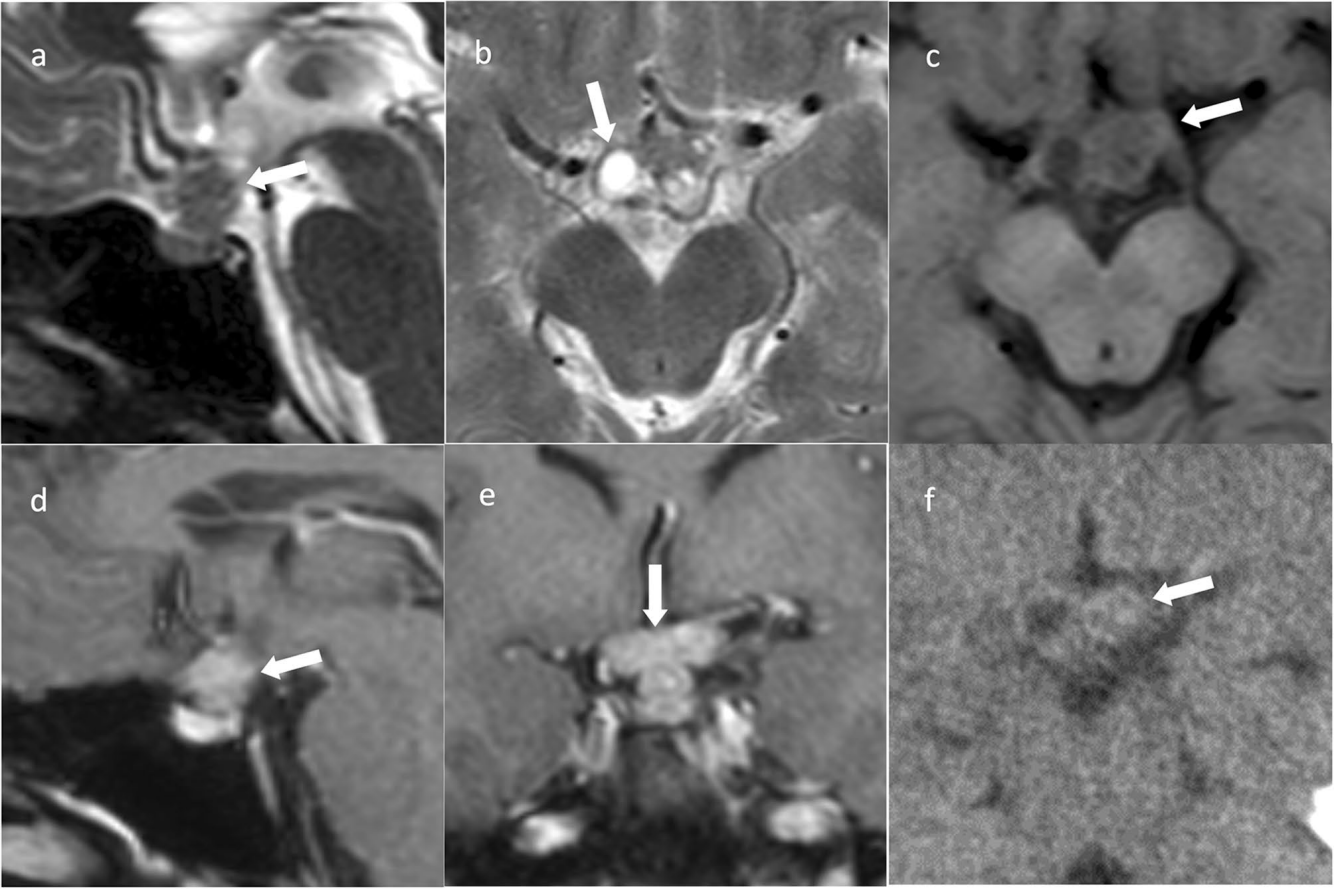
Germinoma. An approximately 30-year-old male with poor vision and no uropathy at the time of magnetic resonance imaging. **a** Sagittal T2WI shows a mass in the upper part of the sella turcica with isointensity compared to gray matter (arrow). **b** Axial T2WI shows a cystic component (arrow). **c** Axial T1WI shows an isointensity mass (arrow). **d** Sagittal contrast-enhanced T1WI shows relatively uniform contrast enhancement (arrow). **e** Coronal contrast-enhanced T1WI shows infiltration of the optic chiasm (arrow). **f** Non-contrast computed tomography shows isodensity (arrow). Germinoma was diagnosed based on craniotomy biopsy. Post-biopsy, steroids were administered, and central diabetes insipidus/arginine vasopressin deficiency (CDI/AVP-D) developed. This was attributed to the presence of an adrenocorticotropic hormone secretory defect, a phenomenon known as masked CDI/AVP-D

Suprasellar germinomas are termed neurohypophyseal germinomas and are found in the hypothalamus, pituitary stalk, and posterior pituitary gland [31]. CT shows high density compared to gray matter, and T1WI shows isointensity compared to gray matter, T2WI shows hypointensity to hyperintensity compared to gray matter, contrast-enhanced T1WI shows a uniform contrast enhancement, and diffusion-weighted imaging (DWI) shows hyperintensity (Fig. 4) [32]. The lesion is predominantly located in the pituitary stalk and posterior pituitary gland, with a high rate of uropathy and loss of signal on T1WI in the posterior pituitary gland, differentiating it from PitNET/pituitary adenoma [32].

## Meningioma

Meningiomas are the most common primary central nervous system tumors, and 5%–10% of all intracranial meningiomas occur in the sellar and parasellar regions. Most meningiomas are WHO grade 1 and benign. Meningiomas are found in adults with a female predominance (male-to-female ratio, 1:3) [7]. Sellar or parasellar meningiomas often occur in the tuberculum, diaphragma, and dorsum sellae, and planum sphenoidale, clinoid processes, and cavernous sinuses [33]. Purely intrasellar meningiomas are rare, develop from the periphery, and extend into the sella turcica [34].

### Imaging findings

**Fig. 5:**
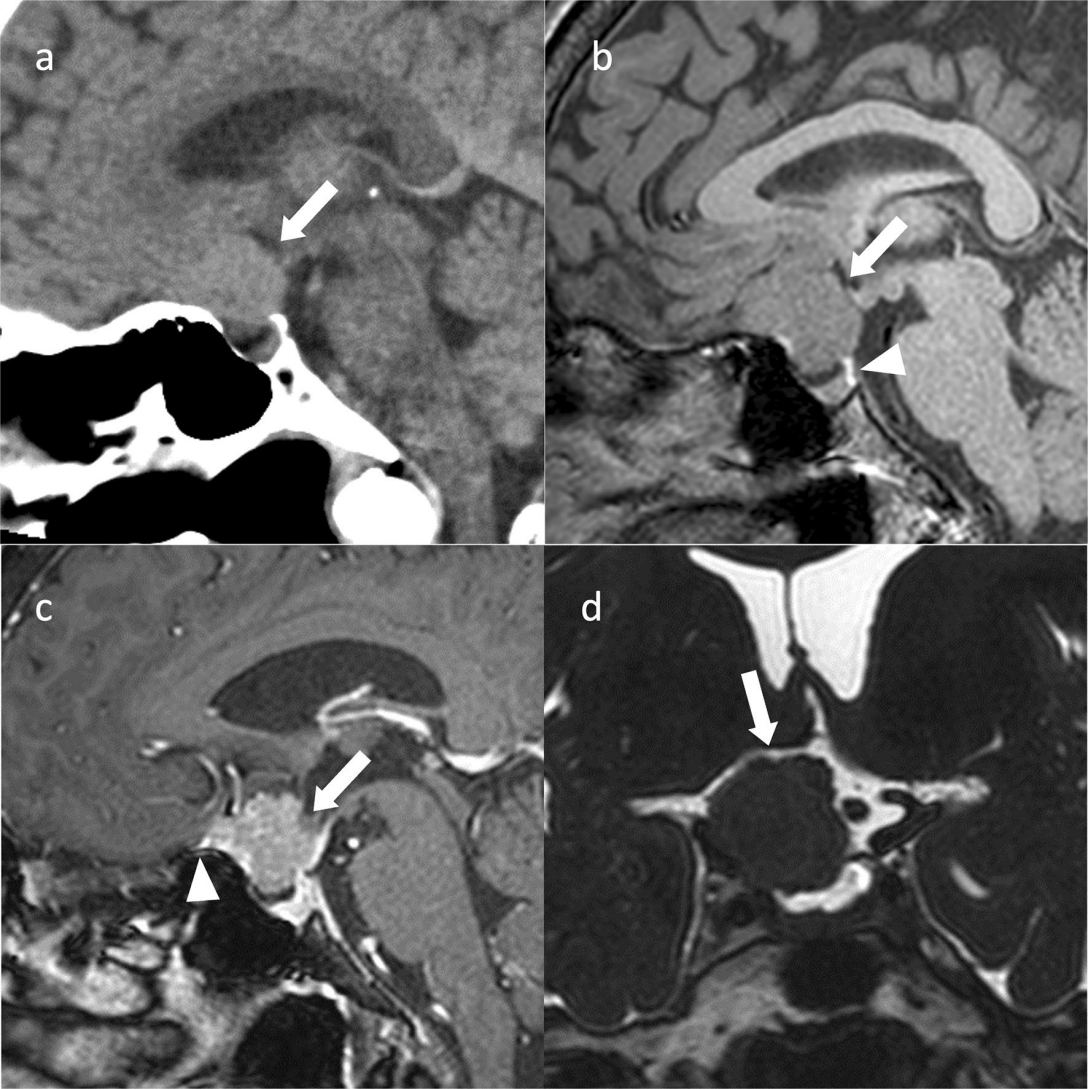
Tuberculum sellae meningioma (meningothelial meningioma). An approximately 60-year-old female with decreased right visual acuity. **a** Sagittal non-contrast computed tomography showing a mildly hyperdensity mass compared to white matter in the suprasellar region (arrow). No obvious thickening of the bone adjacent to the mass is observed. **b** On sagittal T1WI, the mass shows isointensity compared to gray matter (arrow). Normal posterior pituitary gland hyperintensities are observed (arrowhead). **c** Sagittal contrast T1WI shows uniform and strong contrast enhancement (arrow). The dural tail sign is observed anterior to the mass (arrowhead). **d** Coronal heavy T2WI shows compression of the right optic nerve by the mass (arrow). Craniotomy was performed, and the tumor was confirmed as a meningothelial meningioma

Meningiomas usually show isointensity to gray matter on T1WI, slight hyperintensity to hypointensity to gray matter on T2WI, and strong and uniform contrast enhancement, often with a dural tail (Fig. 5) [33]. CT may show calcification within the tumor, bony hyperostosis, or an enlarged sphenoid sinus (pneumosinus dilatans) in adjacent regions [34]. The factors distinguishing it from PitNET/pituitary adenoma are that it is more common in women, presents with a normal (compressed) pituitary gland, rarely shows a snowman shape, and has no sellar dilation, and uniform contrast enhancement is observed, a dural tail is frequently observed, bony hyperostosis is present in adjacent regions, and the ADC is higher than that of PitNET/pituitary adenoma [35].

## Chordoma

Chordomas account for 1–4% of all primary bone tumors, occurring primarily in the axial bone in the skull base region (32%), the sacrococcygeal region (29.2%), the spine (32.8%), and outside the axial bone (6.0%) [36]. Chordomas account for 0.5% of sellar lesions [2]. Skull base chordomas typically arise from the clivus and can extend to the parasellar or suprasellar region [2]. Entirely intrasellar lesions are rare and are recognized as originating from ectopic notochordal tissue located in the sella turcica [37]*.* The mean age of intrasellar chordoma (55.5 years) is older than that of non-intrasellar skull base chordoma (43.3 years) [2].

Chordomas are divided into four subtypes in the 5th edition of the WHO Classification of Endocrine and Neuroendocrine Tumors*:* conventional, chondroid, de-differentiated, and poorly differentiated [2]. Poorly differentiated chordoma has been recognized as a subtype with clinicopathological features characterized by loss of SMARCB1 expression [38] and is newly listed in the 5th edition of the WHO Classification of Endocrine and Neuroendocrine Tumors. It typically occurs in young adults and children, with a median age of 11 years (range: 1–29 years) [39].

### Imaging findings

**Fig. 6:**
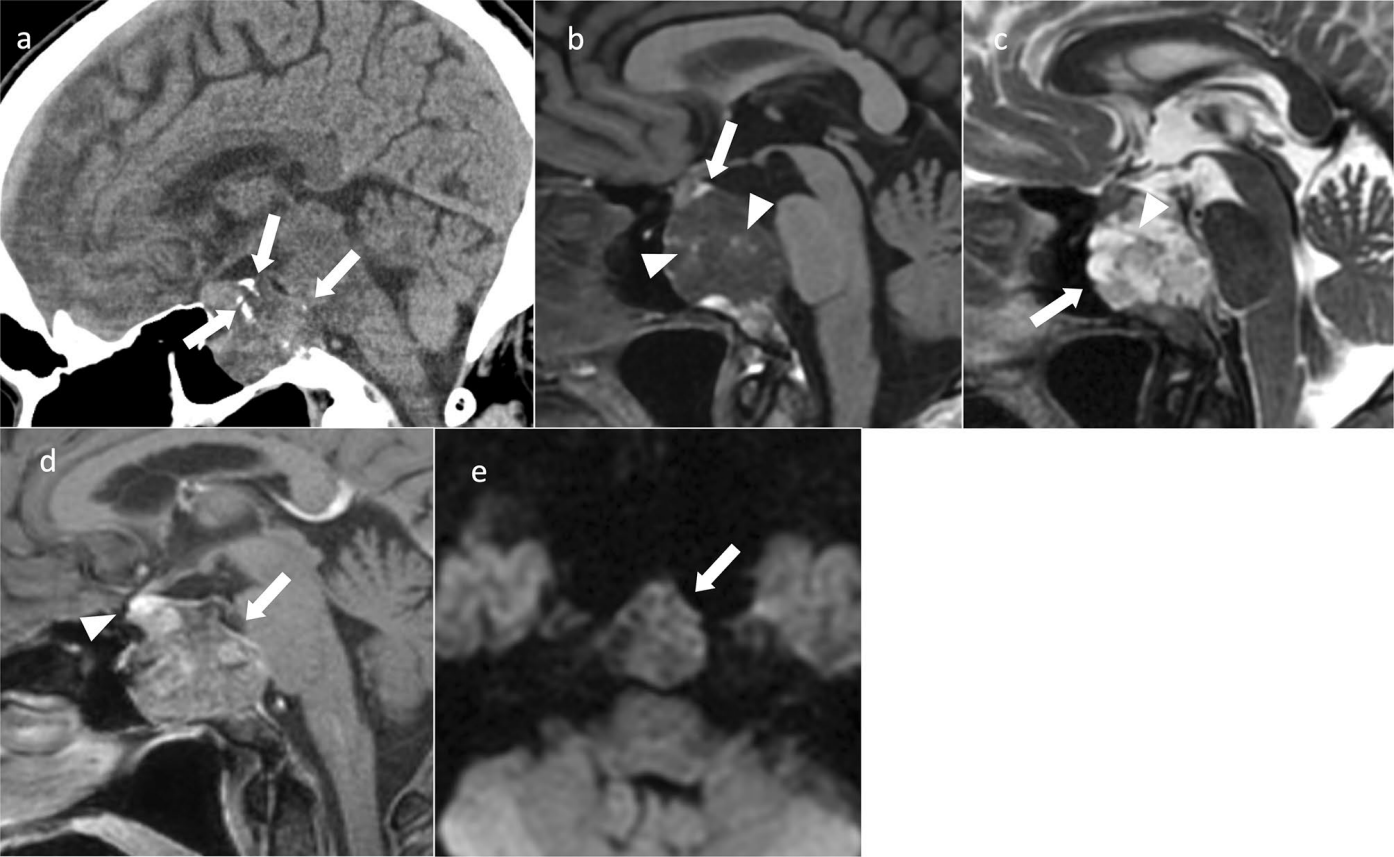
Clivus chordoma (conventional chordoma). An approximately 60-year-old male with polyopia. **a** Sagittal non-contrast computed tomography shows isodensity mass compared to white matter. Some hyperdensity area is observed suggesting sequestered fragments of the destroyed bone (arrows). **b** On sagittal T1WI, the mass shows hypointensity compared to white matter. Some small hyperintensity foci are observed in the mass (arrowhead). Pituitary gland is mildly pushed upward by the mass with normal hyperintensity in the posterior pituitary gland (arrow). **c** On sagittal T2WI, the mass shows marked hyperintensity (arrow) with hypointensity septations (arrowhead). **d** Sagittal contrast-enhanced T1WI shows moderate contrast enhancement in the mass (arrow), weaker than the pituitary contrast enhancement (arrowhead). **e** Diffusion-weighted imaging (DWI) shows isointensity compared to white matter in the mass (mean apparent diffusion coefficient = 1.4 × 10^–3^ mm^2^/s) (arrow)

Chordomas are usually located in the midline [40]. On non-contrast CT, conventional chordoma shows typically well-circumscribed, hyperdensity compared to the neuronal axis, heterogeneous lesions with extensive lytic bone destructions [40]. T2WI typically shows marked hyperintensity reflecting high fluid content of vacuolated cellular components [40] (Fig. 6). Hypointense septations are commonly seen that separate hyperintensity lobules [40]. T1WI shows iso- to hypointensity compared to muscle, but may show small hyperintense foci in the tumor, a finding suggestive of intratumoral hemorrhage or mucus pooling [40, 41]. The majority of conventional chordomas show moderate-to-marked contrast enhancement [40]. The enhancement patterns may present a “honeycomb” appearance formed by areas of low signal intensity within the tumor [40]. Chordomas with non-contrast or minimal contrast may suggest a better prognosis than those that show more marked enhancement [42]. The median ADC of conventional chordomas is about 1.5 × 10^–3^ mm^2^/s [43]. Chondroid chordomas show similar imaging findings to conventional chordomas [44]. Calcifications in the tumor reflecting the cartilage components may be a characteristic of chondroid chordoma, but it is sometimes difficult to distinguish from sequestered fragments of the destroyed bone seen in the conventional chordomas [40]. De-differentiated chordoma shows isointensity to the muscle on T1WI, and hyperintensity on T2WI [44]. This subtype also seems to be difficult to distinguish from conventional chordomas on imaging findings. Poorly differentiated chordoma often shows hypointensity on T2WI compared to conventional chordomas; however, there is a lack of the literature to establish consistent imaging characteristics [44].

Differential diagnoses include chondrosarcoma, metastasis, and invasive PitNET/pituitary adenoma. Chondrosarcomas are usually arising from the petro-occipital fissure away from the midline [43]. Chondrosarcomas, like chordomas, show hyperintensity on T2WI. The median ADC of chondrosarcomas is about 2.0 × 10^–3^ mm^2^/s and it is higher than that of chordomas [43]. Metastases shows hypointensity on T2WI compared to chordomas [43]. Invasive PitNET/pituitary adenoma shows lower mean ADC and hypointensity on T2WI compared to chordomas [45]. And the enhancement peak and maximum contrast-enhancement ratio on dynamic MRI are higher for invasive PitNETs/pituitary adenomas than these values for chordomas [45].

## Metastatic tumors

The prevalence of pituitary metastases varies depending on the method of assessment; 0.4% in radiological, 1% in surgical, and 0.14–28.1% in autopsy series [46]. The most common metastases to the pituitary gland are from lung and breast cancers [47]. Breast cancers with pituitary metastases are often HER2-positive (67%) [48]. Pituitary metastases from breast cancer often appear more than 10 years post-breast cancer diagnosis and present as a single metastasis [48]. Pituitary metastases are most often observed in the posterior lobe and less frequently in the anterior lobe only [49]. This is attributed to the supply of the posterior lobe by the pituitary artery, whereas the anterior lobe is supplied by the portal vasculature [49]. Central diabetes insipidus/arginine vasopressin deficiency (CDI/AVP-D) is often present. Metastasis may present as a dumbbell-shaped tumor within the pituitary sella to the suprasellar region. Although differential diagnosis from PitNET/pituitary adenoma can be difficult, the presence of sellar bone erosion without sellar enlargement may support a diagnosis of pituitary metastasis [50]. PitNET/pituitary adenoma rarely cause CDI/AVP-D. Fluorodeoxyglucose-positron emission tomography can also be used to detect PitNETs/pituitary adenomas; however, its usefulness in distinguishing PitNETs/pituitary adenomas from pituitary metastases is limited [50].

## Lymphoma

Primary central nervous system lymphomas (PCNSL) account for 3% of all intracranial neoplasms [51]. PCNSL lesions are seen in a cerebral hemisphere (38%), thalamus/basal ganglia (16%), corpus callosum (14%), periventricular region (12%), and cerebellum (9%) [51]. Primary pituitary lymphomas are very rare, with about 40 cases reported [52]. Secondary pituitary lymphomas are associated with metastasis or invasion of systemic lymphoma and are also rare accounting for less than 0.5% of all reported pituitary metastases [53]. CDI/AVP-D may occur in approximately 40% of pituitary lymphomas [54]. B-cell lymphoma is the most common cell type of primary pituitary lymphoma, followed by T-cell and NK/T-cell types [52]. The prognosis is worse for the primary form, with TP53 mutations and BCL6-LPP fusions reportedly associated with worse prognosis [55].

### Imaging findings

**Fig. 7:**
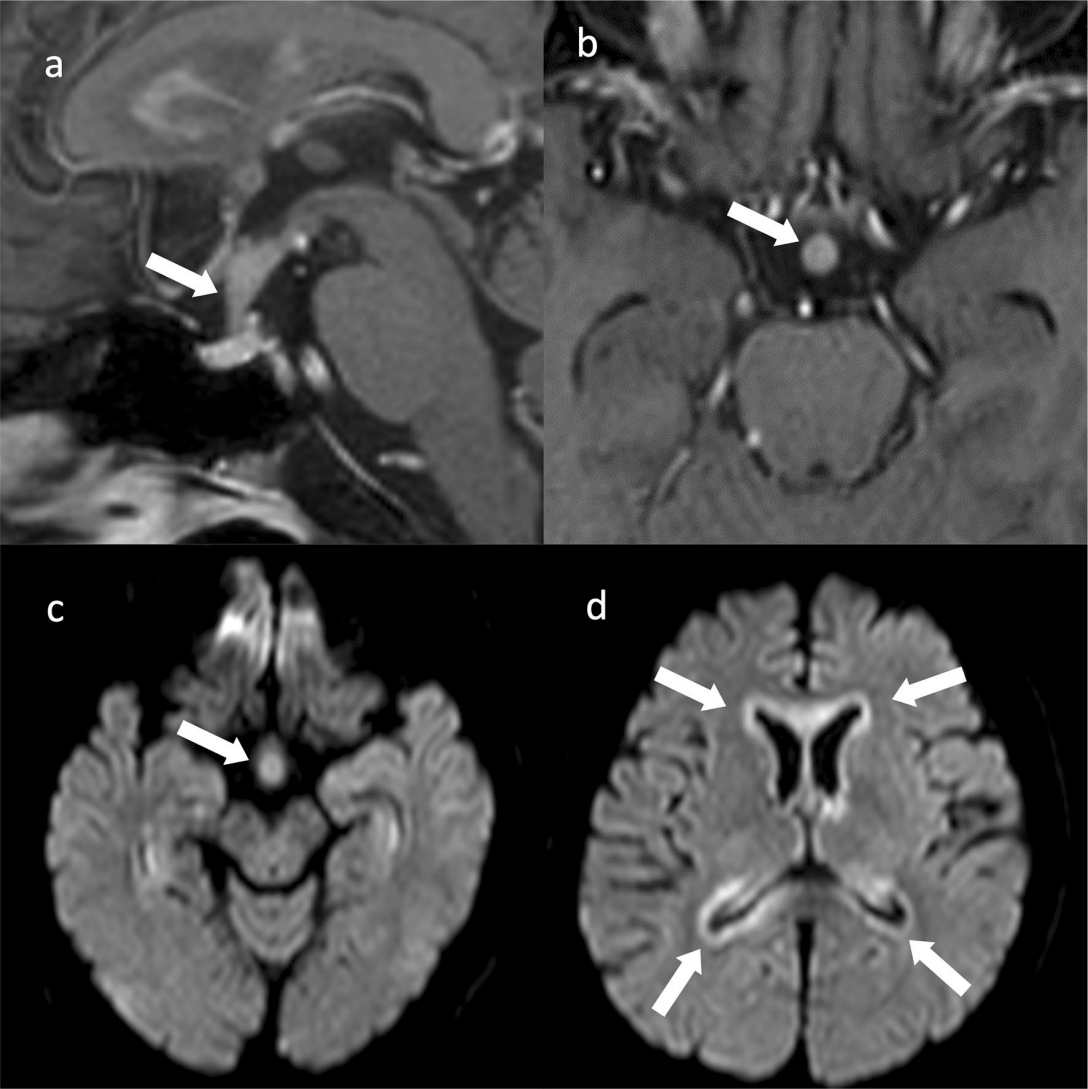
Primary central nervous system lymphoma (PCNSL) involving the pituitary stalk. A 60-year-old male with generalized body malaise. The pituitary stalk is enlarged with contrast enhancement on contrast-enhanced T1WI (arrows) (**a**, **b**) and hyperintensity on diffusion-weighted imaging (DWI) (arrow) (**c**). **d** DWI showing hyperintensity lesion along the ventricular wall DWI showing hyperintensities along the ventricular wall (arrows), which represents hypercellularity. Brain biopsy confirmed a diffuse large B-cell lymphoma

The imaging findings of pituitary lymphoma are isointensity on T1WI and isointensity to hypointensity on T2WI with uniform contrast enhancement [54]. DWI exhibits hyperintensity, reflecting the high cell density of the tumor [56]. The cavernous sinus is involved in approximately 40% of cases. Searching for lymphoma involvement outside the pituitary gland is important (Fig. 7) [56].

## Pituitary incidentaloma

A pituitary incidentaloma is a previously unsuspected pituitary lesion that is discovered in an imaging study performed for an unrelated reason [57]. Pituitary microincidentalomas (lesions < 10 mm) have been observed on CT in 4%–20% or on MRI in 10%–38% of adult patients who underwent cranial imaging studies for reasons other than pituitary disease. Macroincidentalomas (> 10 mm) are rare and are observed on CT in 0.2% of patients and on MRI in 0.16% of patients [57]. Nonfunctioning PitNET/pituitary adenoma (50%–70%) is the most frequent form of pituitary incidentaloma in adults, followed by Rathke’s cleft cysts (13%–18%) and prolactinomas (11%–14.4%) [58]. Rathke’s cleft cysts are more common (67.7%) in children [58].

Guidelines published in 2011 by the American Endocrine Society recommend that patients with pituitary incidentalomas, regardless of size, undergo screening for pituitary function. If lesions are adjacent to the optic nerve or optic chiasm, a visual field examination is recommended. Surgery is recommended for hormone-producing tumors, other than prolactinomas, and for visual field disturbances. MRI follow-up is recommended at 6 months for lesions larger than 10 mm and at 1 year for lesions smaller than 10 mm, followed by progressively less-frequent follow-up if there is no change in lesion size [57]. According to the Japanese Guidelines for Brain Dock 2019, if a pituitary mass is detected, pituitary and related peripheral hormone levels should be measured to rule out pituitary hormone hypersecretion or hypopituitarism, even if patients are asymptomatic. Baseline levels of growth hormone (GH), insulin-like growth factor 1 (IGF-1), prolactin (PRL), ACTH, cortisol, thyroid-stimulating hormone (TSH), free T3 and T4, follicle-stimulating hormone, luteinizing hormone, testosterone, and estradiol (E2) are measured. When a substantial mass is discovered in the pituitary region, surgery (mainly transsphenoidal) is recommended if suprasellar extension (contact with or elevation of the optic tract) is observed; however, pituitary hyperplasia should be ruled out. Small lesions that do not contact the optic tract should be followed up with MRI and the aforementioned hormone levels, initially twice every 6 months and annually thereafter. If cystic lesions are discovered in the pituitary region, follow-up MRI and hormone level measurements are performed twice every 6 months at first, and annually thereafter. Pituitary incidentaloma treatment is anticipated to undergo future changes concomitant with the name change from pituitary adenoma to PitNET [59].

## Pathologies requiring differentiation from tumors

### Pituitary abscess

The two main abscess types are primary, in which infection occurs in the normal pituitary gland, and secondary, in which infection occurs in pituitary lesions, such as PitNET/pituitary adenoma, Rathke’s cleft cysts, and craniopharyngioma [60].

Symptoms, such as headaches, anterior pituitary hypopituitarism, CDI/AVP-D, and visual field abnormalities, are often caused by pituitary masses. Under 50% of patients present with inflammatory symptoms such as fever and meningitis [61].

#### Imaging findings

**Fig. 8:**
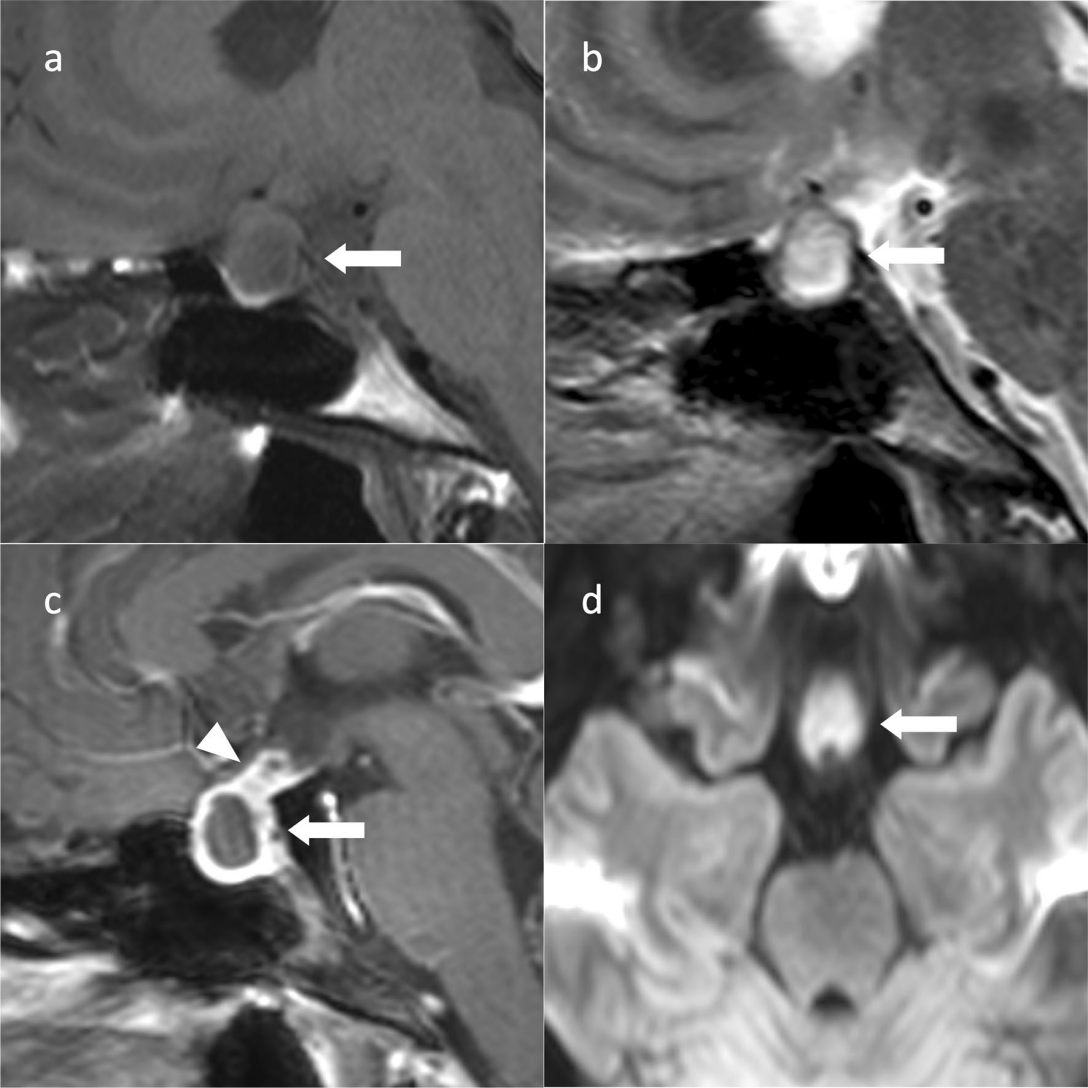
Pituitary abscess. An approximately 20-year-old female with headaches and nausea. Sagittal T1WI (**a**) and sagittal T2WI (**b**) show a cystic mass within the sellar turcica to the suprasellar region (arrow). Sagittal T2WI (**b**) failed to show hypointense regions of the peripituitary region (parasellar T2-dark sign). **c** Sagittal contrast-enhanced T1WI shows contrast enhancement of the cyst wall (arrow) with an enlarged pituitary stalk (arrowhead). **d** Diffusion-weighted imaging (DWI) shows marked hyperintensity inside (mean apparent diffusion coefficient = 0.5 × 10^–3^ mm^2^/s) (arrow)

Imaging reveals a cystic abscess within the pituitary gland and a pituitary mass. Depending on the nature of the abscess interior, T2WI may exhibit high to equal intensity and T1WI may show equal to low intensity [62]. A rim-like contrast enhancement has been observed [62]. Inside the cyst shows hypointensity on contrast-enhanced T1WI, but hyperintense flocculent or cotton-like foci may also be present [63]. The abscess area has a hyperintensity lesion on DWI (Fig. 8) [64]. Thickening of the surrounding dura mater may be observed. Half of the cases are accompanied by thickening of the pituitary stalk [63]. The normal pituitary stalk is 2.32 ± 0.39 mm at the pituitary insertion and 3.25 ± 0.43 mm at the level of the optic chiasm, with a smaller diameter at the inferior end than at the superior end [65]. Pituitary abscess is accompanied by thickening of the pituitary stalk, predominantly at the inferior end, with the diameter of the lower end of the stalk can being larger than that of the upper end [66].

DWI is useful for differentiating pituitary abscess from cystic PitNET/pituitary adenoma. Non-hemorrhagic infarction-type pituitary apoplexy may be difficult to differentiate because of the rim-like contrast enhancement and the possibility of hyperintensity on DWI. However, pituitary apoplexy has a more acute onset, and pituitary abscesses often show thickening of the pituitary stalk, predominantly in the inferior region.

### Hypophysitis

Hypophysitis can be divided into two types: primary and secondary. Primary hypophysitis is further subdivided into lymphocytic, granulomatous, and xanthomatous types, with lymphocytic types being the most common [67]. Lymphocytic hypophysitis is restricted to the anterior pituitary gland and is thought to occur during the perinatal period. Hypophysitis has been reported in the anterior lobe, posterior lobe or pituitary stalk, or in the entire pituitary gland, referred to as lymphocytic adenohypophysitis, lymphocytic infundibuloneurohypophysitis, and lymphocytic panhypophysitis, respectively [68, 69]. Recent reports indicate that perinatal cases are less common and males may be affected [70]. Secondary causes include drug-induced effects, infectious diseases (tuberculosis, syphilis, etc.), sarcoidosis, granulomatosis with polyangiitis, Langerhans cell histiocytosis, and IgG4-related diseases [67]. Although immune checkpoint inhibitors (cytotoxic T-lymphocyte-associated antigen-4 [CTLA-4] inhibitors, programmed death-1 [PD-1] inhibitors, and programmed death-ligand-1 [PD-L1] inhibitors) have been increasingly used in recent years to treat various malignancies (melanoma, pancreatic cancer, and non-small cell lung cancer), pituitary inflammation caused by immune checkpoint inhibitors has been reported [71, 72]. The probability of hypophysitis in conjunction with immune checkpoint inhibitor use is as follows: combination therapy (nivolumab + ipilimumab), 6.4% and 3.2%; CTLA-4 inhibitors, 0.4%; and PD-1 inhibitors, PD-L1 inhibitors, and CTLA-4 inhibitors, < 0.1% [73]. Moreover, anti-PIT-1 antibody-associated hypophysitis, an acquired decrease in GH, PRL, and TSH (hormones of the PIT-1 group) associated with anti-PIT-1 antibodies associated with thymoma and malignancy, has been reported [74].

### Imaging findings

**Fig. 9:**
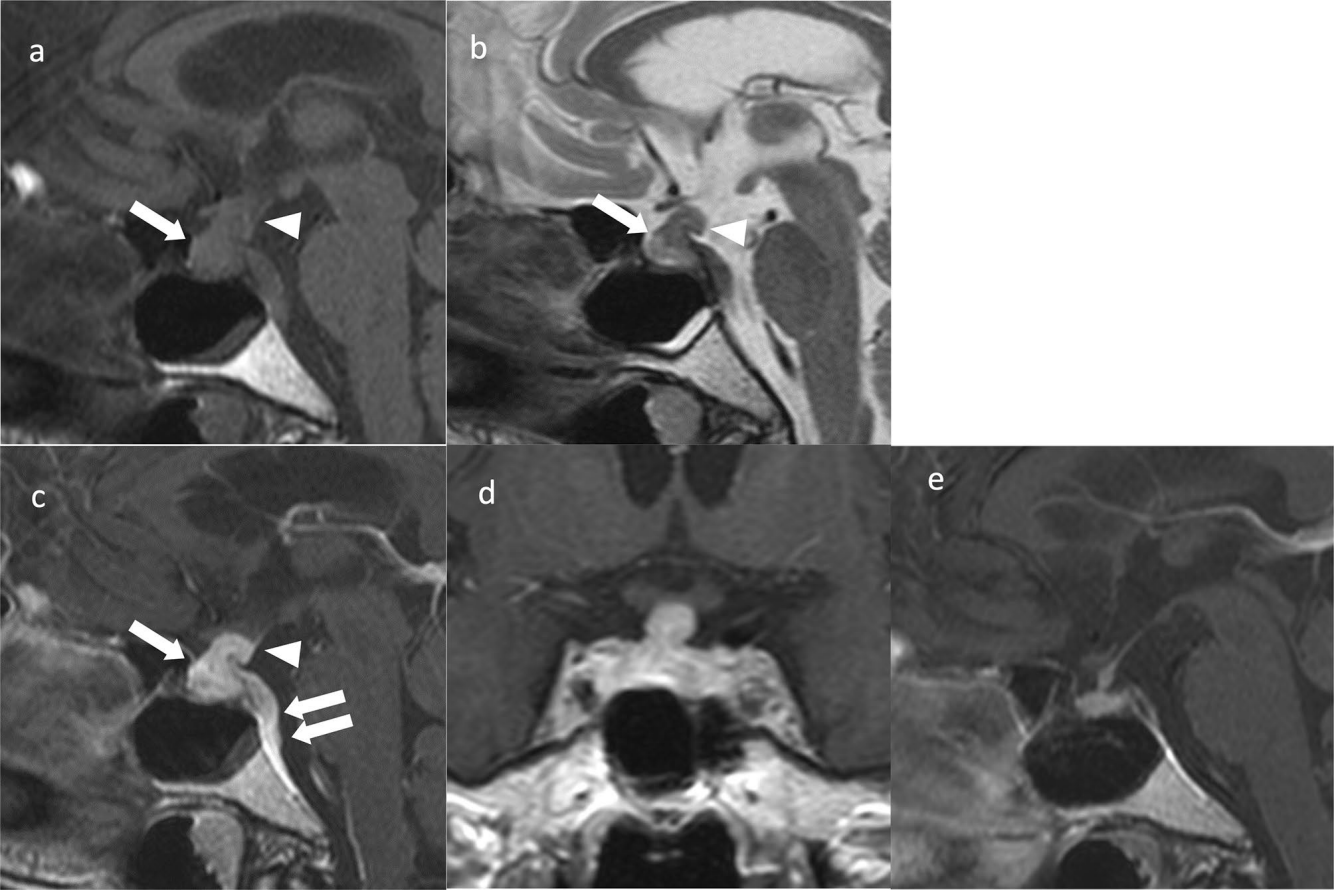
Lymphocytic hypophysitis. An approximately 80-year-old female with left oculomotor nerve palsy. Sagittal T1WI (**a**) and sagittal T2WI (**b**) show an enlarged pituitary gland (arrows) and pituitary stalk (arrowheads). Sagittal T1WI (**a**) shows no hyperintensity in the posterior pituitary. **c** Sagittal contrast T1WI shows comparative homogeneous contrast effects of the pituitary gland (arrow) and stalk (arrowhead). A thickened dura mater is observed on the dorsal surface of the clivus (double arrow). **d** Coronal contrast-enhanced T1WI shows a symmetrical enlarged pituitary gland and stalk. **e** Sagittal contrast-enhanced T1WI after 3 months of oral steroid treatment shows a reduction in the size of the pituitary gland and stalk. Dural thickening also improved

Hypophysitis images are similar for each type. Symmetrical enlargement of the pituitary gland and stalk, uniform contrast enhancement, and loss of hyperintensity in the posterior pituitary gland on T1WI are observed [75]. Thickening of the surrounding dura mater (dural tail) may be observed (Fig. 9) [75]. Hypointense regions on T2WI of the peripituitary region (parasellar T2-dark sign) may also be observed [76]. Dynamic contrast-enhanced MRI may show loss or delay of contrast enhancement of the posterior pituitary gland in the early phase and delay of contrast enhancement throughout the pituitary gland [77].

Diffuse basisphenoid enhancement may be seen in granulomatous hypophysitis, a finding that has been reported as absent in PitNETs/pituitary adenomas [78]. In immune checkpoint inhibitor-induced pituitary inflammation, the anterior lobe of the pituitary gland exhibits geographic hypo-enhancing lesions on contrast-enhanced MRI, and these lesions show hypointensity on T2WI, reflecting fibrosis [79]. In anti-PIT-1 antibody-associated hypophysitis, the pituitary gland is often mildly atrophic or normal [80]. If hypophysitis is caused by IgG4-related disease, lacrimal gland enlargement, infraorbital nerve enlargement, intraorbital inflammatory pseudotumors, and hypertrophic pachymeningitis may be observed on cranial MRI [81].

The scoring method proposed by Gutenberg et al. is useful for distinguishing pituitary inflammation from macroadenoma (sensitivity, 92%; specificity, 99%) (Table 3) [82].

**Table 3:** Scores to distinguish autoimmune hypophysitis from nonsecreting PitNET/pituitary adenoma

Age (year) ≤ 30	− 1
Relation to pregnancy: yes	− 4
Pituitary volume (cm^3^) ≥ 6	+ 2
Gd enhancement type: medium or high	− 1
Gd enhancement features: Heterogeneous	+ 1
Asymmetric	+ 3
Posterior pituitary bright spot: lost	− 2
Stalk size: enlarged	− 5
Mucosal thickening: present	+ 2

Among the nine items, a total score of less than 1 indicates pituitary inflammation and 1 or more indicates PitNET/pituitary adenoma (sensitivity 92%; specificity 99%)

PitNET: pituitary neuroendocrine tumor; Gd: gadolinium

Reproduced from [82] in part with permission from American Society of Neuroradiology

#### Pituitary hyperplasia

The pituitary gland can physiologically enlarge during the neonatal period, puberty, pregnancy, postpartum, and menopause, and is often approximately 3–8 mm in adolescent and adult males and 4–10 mm in adolescent and adult females [83].

Primary hypothyroidism is the most common cause of pituitary hyperplasia. Other possible causes include physiological factors, primary hypogonadism (Klinefelter syndrome and Turner syndrome), polycystic ovary syndrome (PCOS), primary hypoadrenocorticism (Addison’s disease), and antipsychotic drugs [84].

The mechanism of pituitary hyperplasia due to primary hypothyroidism is that decreased thyroid hormones stimulate the secretion of thyroid-stimulating hormone-releasing hormone (TRH) in the hypothalamus, leading to hyperplasia of the thyrotroph cells. TRH is also a weak stimulator of lactotroph cells and can cause high prolactin levels [85]. These changes are reversible and return to normal with thyroid hormone replacement. Conversely, a marked decrease in thyroid function, even after 3 weeks, can cause significant pituitary gland enlargement [86].

### Imaging findings

**Fig. 10:**
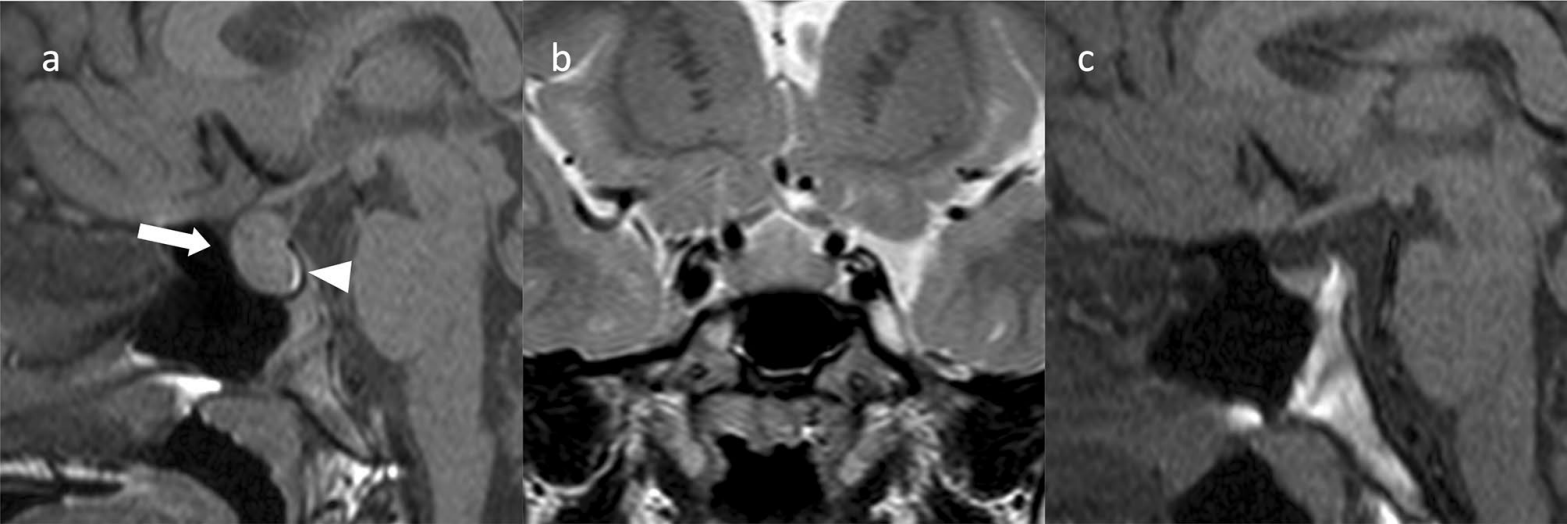
Pituitary hyperplasia associated with primary hypothyroidism. A 10-year-old male with short stature and free T4 levels at 0.52 ng/dL (mildly low) and thyroid-stimulating hormone levels at 794.8 μIU/mL (high). **a** Sagittal T1WI shows pituitary enlargement. The pituitary body is 13-mm high (arrow), with normal hyperintensity in the posterior pituitary gland (arrowhead). **b** Coronal T2WI shows symmetric enlargement of the anterior pituitary gland. **c** The patient was treated using thyroid hormone replacement therapy; 10 years post-treatment, the pituitary gland was normal in size on T1WI

Hyperplasia often shows homogeneous intensity and contrast enhancement on MRI (Fig. 10) [87]. PitNETs/pituitary adenomas can be differentiated by their heterogeneous intensity, T1WI, and contrast-enhanced images that show hypointensity compared with those of the normal pituitary gland [87]. However, hyperplasia is often difficult to distinguish from PitNET/pituitary adenomas using imaging. It is important to search for endocrine factors (especially hypothyroidism) that may cause hyperplasia.

During late pregnancy and the postpartum period, the anterior pituitary gland is physiologically enlarged and may show hyperintensity on T1WI [88, 89]. This should not be mistaken for pituitary hemorrhage or PitNET/pituitary adenoma with hemorrhage.

#### Rathke’s cleft cyst

Rathke’s cleft cysts are non-malignant cystic lesions derived from the remnants of Rathke’s pouch. The cysts occur between the anterior and posterior lobes of the pituitary gland in the sella turcica or suprasellar region. They are rarely symptomatic and are often discovered incidentally [90]. The frequency of Rathke’s cleft cysts larger than 2 mm increases with age [91]. The frequency of these cysts in children (1.2% [< 15 years of age]) is purportedly lower than that in adults and is similar to the frequency of Rathke’s cleft cysts in those aged 10–29 years. However, the frequency of the cysts during childhood may have been underestimated. When these cysts enlarge, the surrounding structures (optic chiasm, hypothalamus, and pituitary gland) can be damaged, causing symptoms, such as headache, anterior hypopituitarism, and abnormal vision [5].

### Imaging findings

**Fig. 11:**
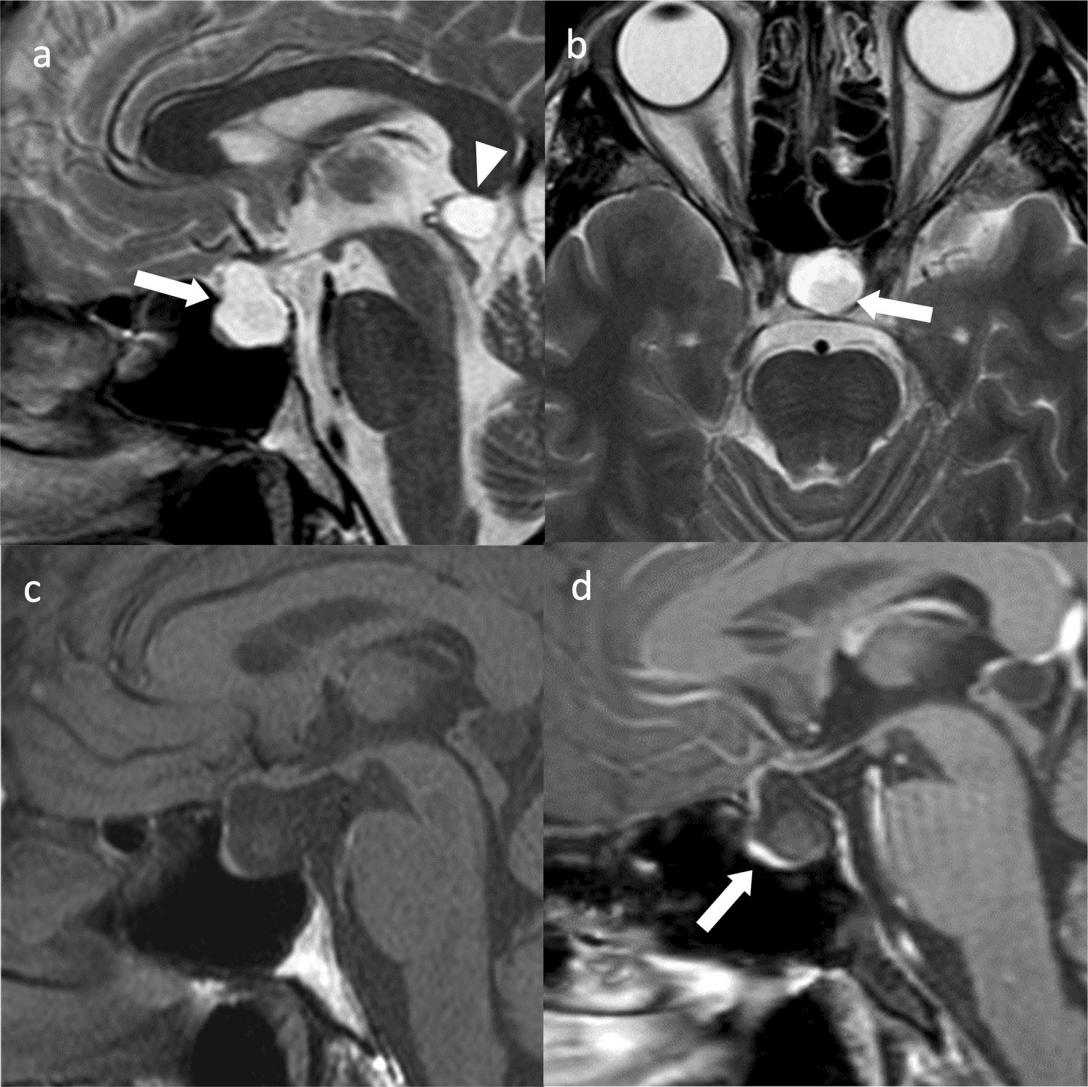
Rathke’s cleft cyst. An approximately 40-year-old female with central diabetes insipidus/arginine vasopressin deficiency. **a** Sagittal T2WI shows a cystic mass in the sella turcica extending to the suprasellar region (arrow). An incidental pineal cyst is observed in the pineal gland (arrowhead). **b** Axial T2WI shows a mild hypointense nodular area (waxy nodule) inside the cyst. **c** Sagittal T1WI shows no hyperintensity in the normal posterior pituitary gland. **d** Sagittal contrast-enhanced T1WI shows an area with contrast enhancement anteriorly and inferiorly within the sella turcica (arrow), which is considered a compressed anterior pituitary lobe

Rathke’s cleft cysts often show hyperintensity on T1WI [92]. The intensity of the cyst is influenced by the nature of its contents, particularly the protein concentration [93]. Characteristic intracystic nodules reflecting waxy component (so-called waxy nodule) with hyperintensity on T1WI and hypointensity on T2WI may be observed within the cyst (Fig. 11) [94, 95]. The cyst wall usually has non-contrast enhancement but may have thin contrast enhancement due to inflammation or squamous metaplasia of the wall [11]. The compressed pituitary gland may also be contrasted and appear as a cyst wall [96]. Contrast-enhanced three-dimensional fluid-attenuated inversion recovery MRI may show less-contrast enhancement on the compressed pituitary gland than that in regular contrast-enhanced T1WI and is useful in differentiating Rathke’s cleft cyst from PitNET/pituitary adenoma with cystic degeneration and adamantinomatous craniopharyngioma [97, 98].

Diagnostic differentiation from PitNET/pituitary adenoma with cystic degeneration can be achieved as follows. The presence of a fluid–fluid level, a hypointense rim on T2WI, an off-midline location, septation, and intensity change of the lesion are more common in PitNETs/pituitary adenomas than those in Rathke’s cleft cysts. An intracystic nodule in the lesion is observed significantly more often in Rathke’s cleft cysts than in PitNET/pituitary adenomas [94].

#### Arachnoid cyst

Arachnoid cysts account for approximately 1% of all intracranial masses [99]. Approximately half of intracranial arachnoid cysts occur in the middle cranial fossa [100]. Arachnoid cysts above the sella turcica account for 10% [101] and those within the sella turcica account for 3% [99]. Common symptoms include visual disturbances, headache, and endocrine dysfunction.

The mechanism of arachnoid cystogenesis in the sella turcica is purportedly the formation of arachnoid cysts on the diencephalic membrane itself, which forms the upper surface of the Lilliquist’s membrane (the arachnoid structure between the dorsum of the sella and the mastoid body [102]), or by enlargement of the interpeduncular cistern caused by impaired cerebrospinal fluid (CSF) passage through the Lilliquist’s membrane [101].

One hypothesized mechanism of arachnoid cysts within the sella turcica is that arachnoid cysts arise in this region from a diverticulum that develops between the arachnoid layers and progressively enlarges [103]. This diverticulum either descends through the diaphragm or develops initially within a subdiaphragmatic cistern [103]. Another hypothesis is that the arachnoid structure and CSF enter through a relatively large opening in the sella diaphragm, causing a ball-valve condition between the sella diaphragm and stretched pituitary gland, resulting in a cystic appearance [104].

### Imaging findings

**Fig. 12:**
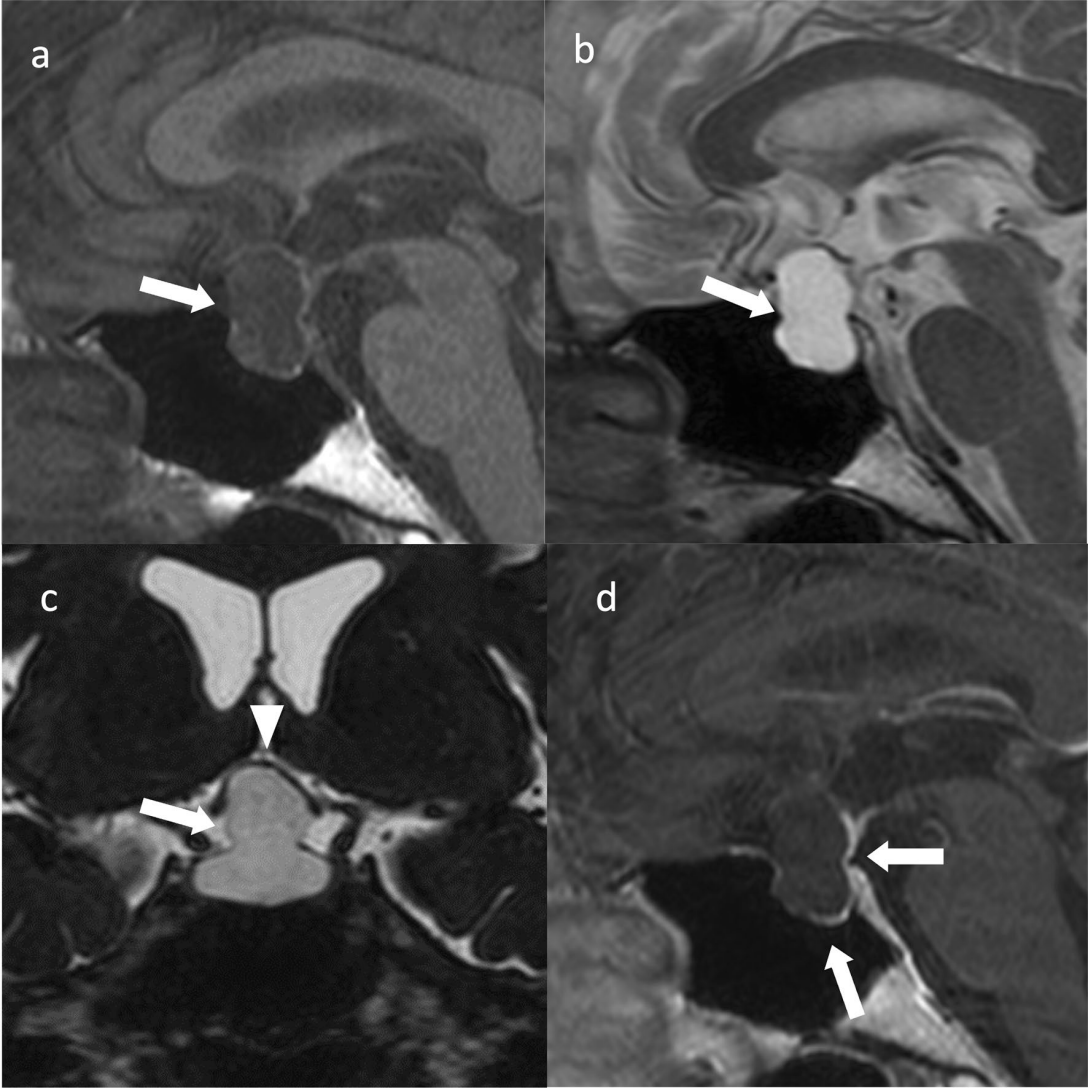
Arachnoid cyst within the sella turcica. An approximately 60-year-old female with 1/4 auricular hemiblindness. **a** Sagittal T1WI shows a hypointensity mass within the sella turcica extending to the suprasellar region (arrow). **b** Sagittal T2WI shows a hyperintensity cystic mass (arrow). **c** Coronal heavy T2WI shows a slightly lower signal than the cerebrospinal fluid signal in the cyst (arrow), with a compression of the optic chiasm (arrowhead). **d** Sagittal contrast T1WI shows an area with contrast enhancement posterior to the base of the cyst wall (arrow), purportedly a stretched pituitary gland

T1WI and T2WI show a thin-walled cyst with a homogeneous signal equal to that of the CSF [104]. Arachnoid cysts within the sella turcica may be slightly hyperintense on T1WI [104]. Arachnoid cysts within the sella turcica can be difficult to distinguish from Rathke’s cleft cysts, but arachnoid cysts tend to push the pituitary gland posteriorly, whereas Rathke’s cleft cysts push it anteriorly (Fig. 12) [99].

#### Aneurysm

**Fig. 13:**
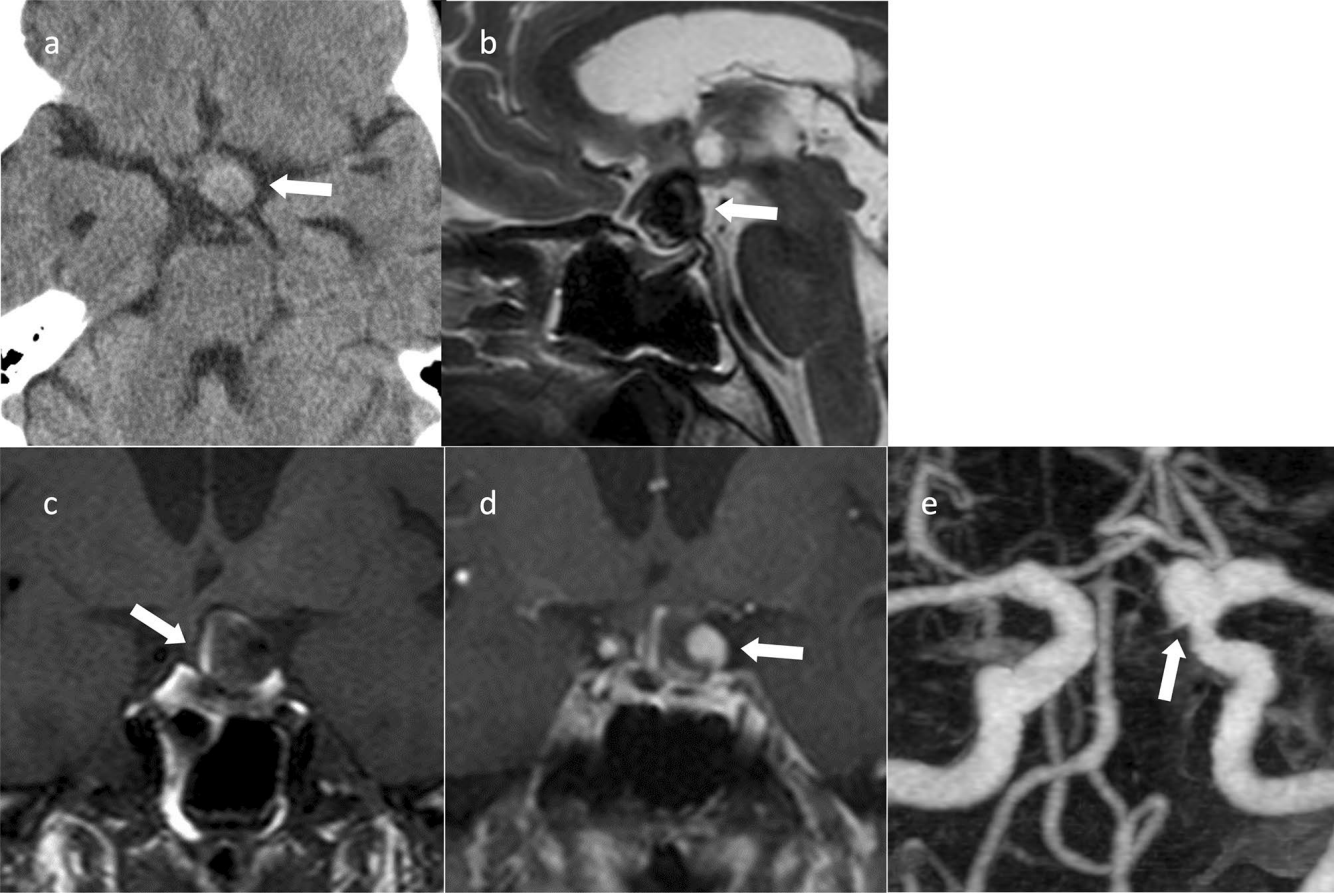
Internal carotid-posterior communicating artery aneurysm with partial thrombosis. An approximately 70-year-old female with headache. **a** Computed tomography shows a high-density mass in the suprasellar region (arrow). **b** Sagittal T2WI shows a hypointensity mass above the sella turcica (arrow). **c** Coronal T1WI shows hypointensity in the mass but with a hyperintense area at the limbus, indicating a thrombus (arrow). **d** Coronal contrast-enhanced T1WI shows a nodular area with contrast enhancement in the mass (arrow). **e** Magnetic resonance angiography shows an aneurysm in the posterior communicating artery of the right internal carotid artery (arrow)

Sellar and parasellar aneurysms can mimic suprasellar/parasellar masses and PitNET/pituitary adenomas. CT and MR angiography aid in diagnosis. The aneurysm is hypointense on T2WI due to flow void [105]. Many aneurysms show thrombosed areas that produce hyper- or heterogeneously intense signals on T1WI. The intensity can also be altered by calcification, lamellated blood degradation products, and flow-related signals (Fig. 13) [7].

Intracranial aneurysms coexist with PitNETs/pituitary adenomas in 2.3% to 6.9% of patients, a prevalence greater than that of the average population [33]. For GH-secreting adenomas, possibly because of the effect exerted by chronic excessive IGF-1 levels on cerebral vascular walls [106], cerebral aneurysms in close proximity to pituitary aneurysms are at risk of rupture during transnasal transsphenoidal surgery if not identified preoperatively and even after treatment, as the supporting power of the tumor weakens. Treatment of cerebral aneurysms should be performed prior to treatment of pituitary aneurysms [107]. It is important to identify and note the presence or absence of a cerebral aneurysm in the vicinity of a pituitary aneurysm (especially GH-secreting adenomas) preoperatively.

## Conclusions

Imaging, particularly MRI, plays an important role in the diagnosis of pituitary-region diseases. For definitive diagnoses, imaging findings, disease location, the lesion’s relationship with the adjacent structures, the nature of the disease, and clinical features must be considered. The 5th editions of the WHO classifications made major changes to the classification of pituitary adenomas, the most common pituitary tumors, and has included several classification changes for other tumors. Radiologists should consider the changes in the 5th editions of the WHO classifications when preparing imaging reports.
