# Correction to: Imaging of pituitary tumors: an update with the 5th WHO Classifications—part 2. Neoplasms other than PitNET and tumor-mimicking lesions

**DOI:** 10.1007/s11604-023-01418-x

**Published:** 2023-04-11

**Authors:** Taro Tsukamoto, Yukio Miki

**Affiliations:** Department of Diagnostic and Interventional Radiology, Graduate School of Medicine, Osaka Metropolitan University, 1-4-3 Asahi-Machi, Abeno-Ku, Osaka, 545-8585 Japan

**Correction to: Japanese Journal of Radiology** 10.1007/s11604-023-01407-0

In the original publication in section Pituitary blastoma, the sentence “DICER1 mutation-associated….” Should read as:

DICER1 mutation-associated tumors include pleuropulmonary blastoma, cystic nephroma, ovarian Sertoli–Leydig cell tumor, ciliary body medulloepithelioma, nasal chondromesenchymal hamartoma, sarcomas of the cervix, kidneys, cerebrum, pituitary blastoma, and pineoblastoma, among others [19].

Part a of Fig. 1 caption should read as:

a Sagittal T2WI shows a multifocal cystic mass above the sella turcica (arrow). The pituitary gland is observed within the sellar turcica

Part b of Fig. 6 caption should read as:

b On sagittal T1WI, the mass shows hypointensity compared to white matter. Some small hyperintensity foci are observed in the mass (arrowhead). Pituitary gland is mildly pushed upward by the mass with normal hyperintensity in the posterior pituitary gland (arrow).

Fig. 13 caption should read as:

a Computed tomography shows a high-density mass in the suprasellar region (arrow). b Sagittal T2WI shows a hypointensity mass above the sella turcica (arrow). c Coronal T1WI shows hypointensity in the mass but with a hyperintense area at the limbus, indicating a thrombus (arrow). d Coronal contrast-enhanced T1WI shows a nodular area with contrast enhancement in the mass (arrow). e Magnetic resonance angiography shows an aneurysm in the posterior communicating artery of the right internal carotid artery (arrow).

In References, Ref. 2 should read as:

2. WHO Classification of Tumours Editorial Board. Endocrine and Neuroendocrine tumours [Internet]. Lyon (France): International Agency for Research on Cancer; 2022 [cited 2022 Nov 1] (WHO classification of tumours series, 5th ed.; vol. 10). https://tumourclassification.iarc.who.int/chapters/53.

The original publication has been corrected.

